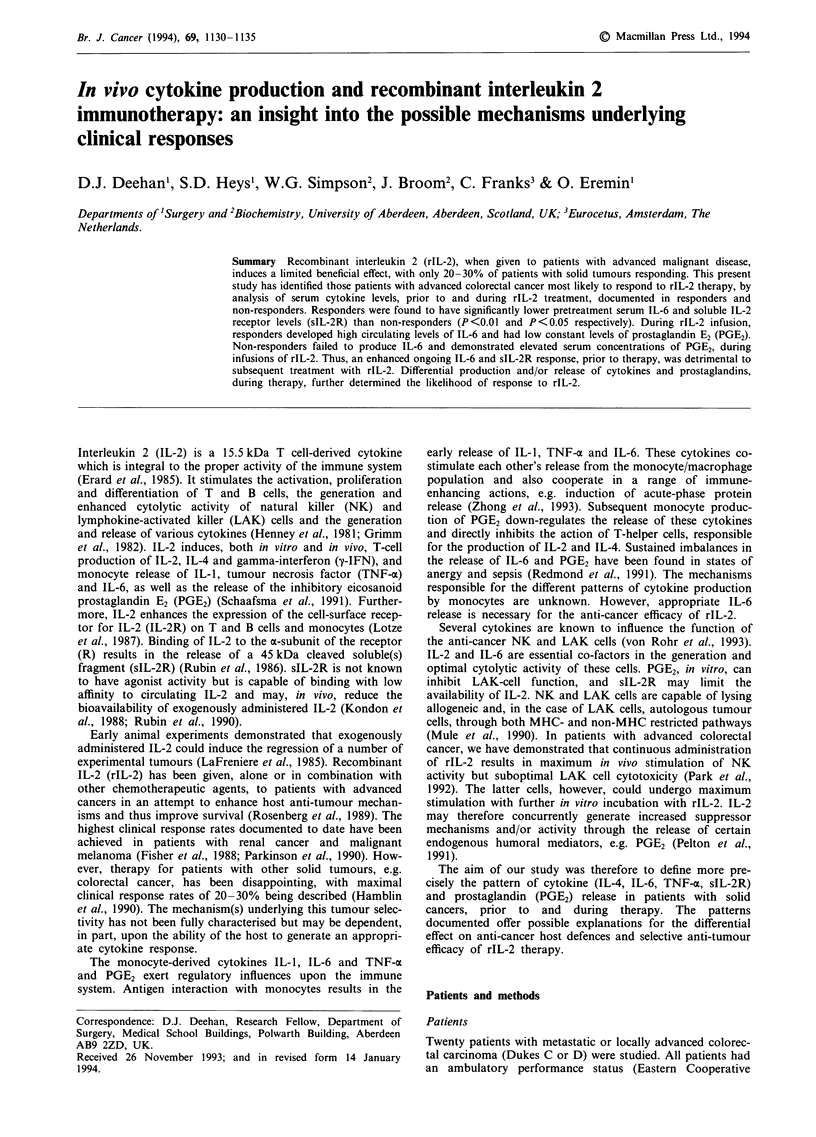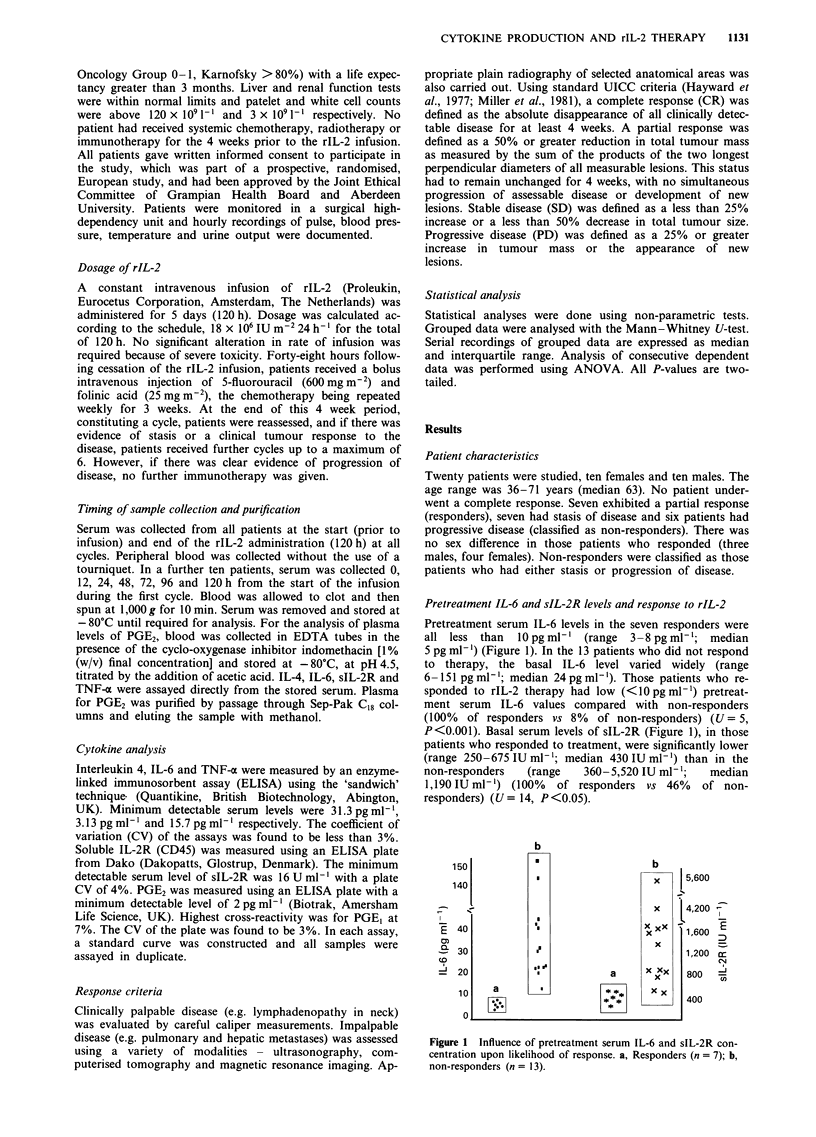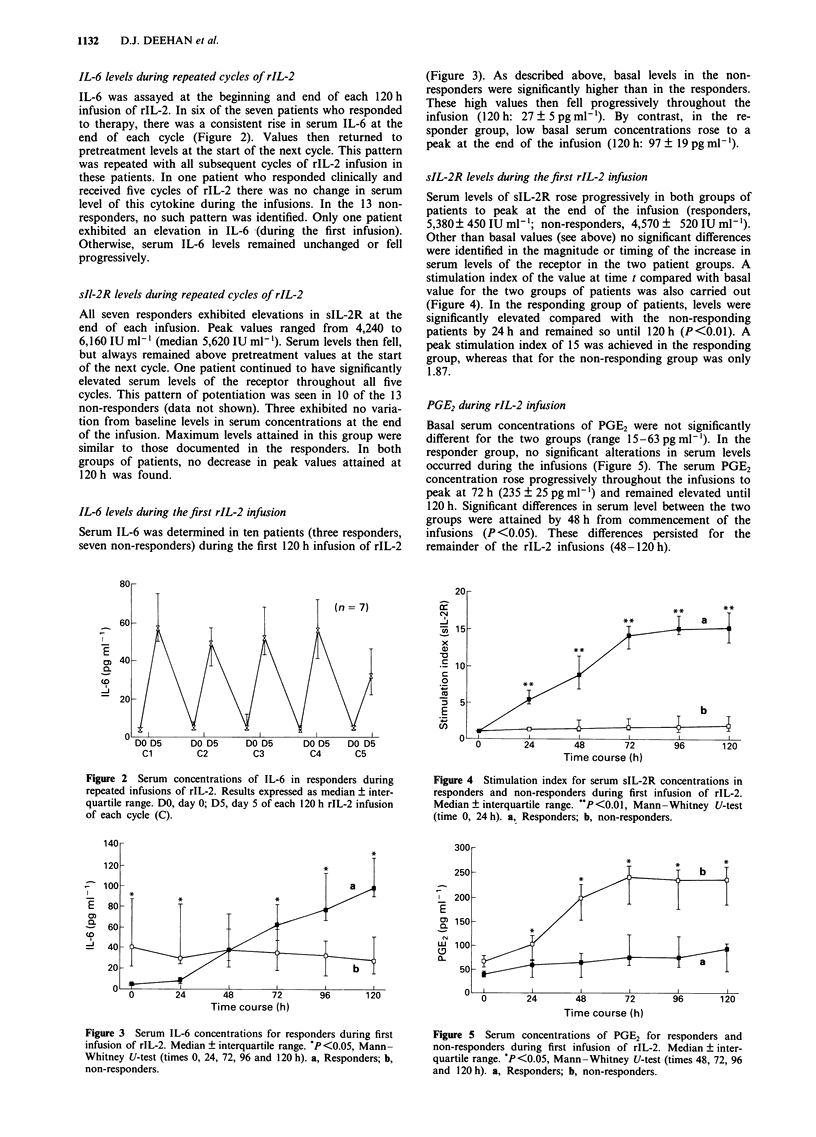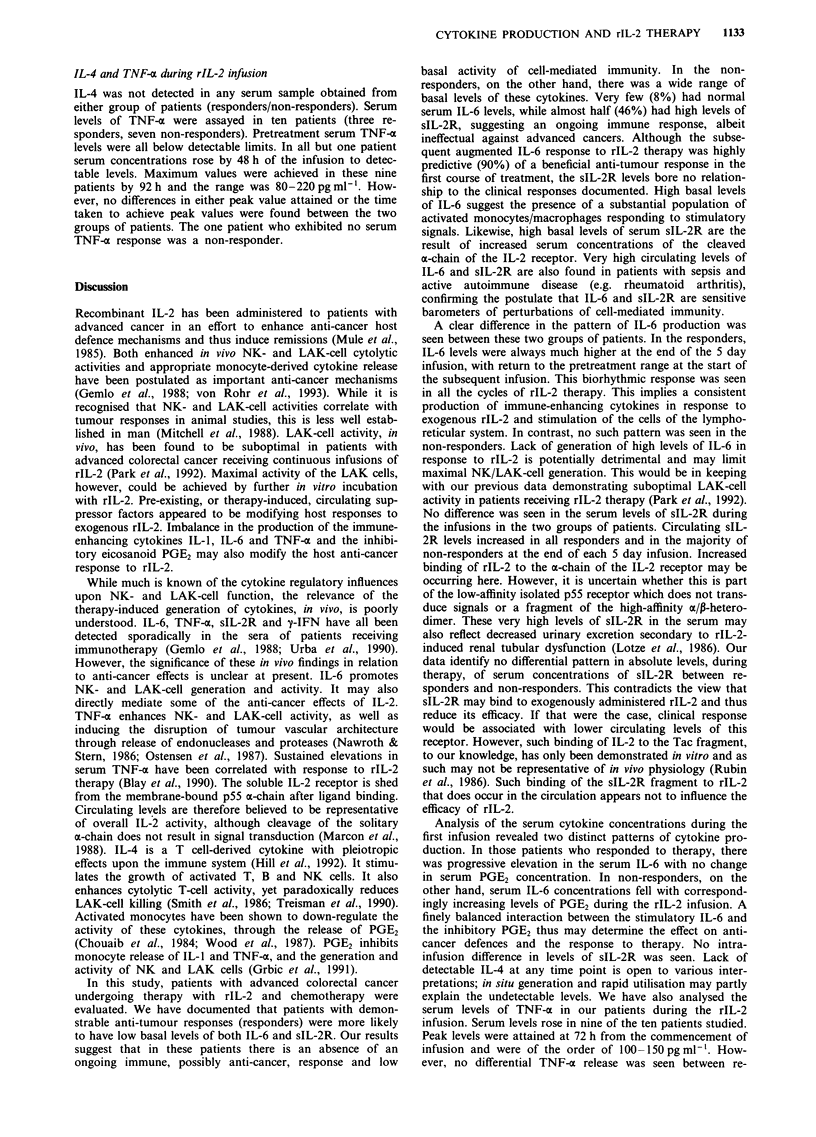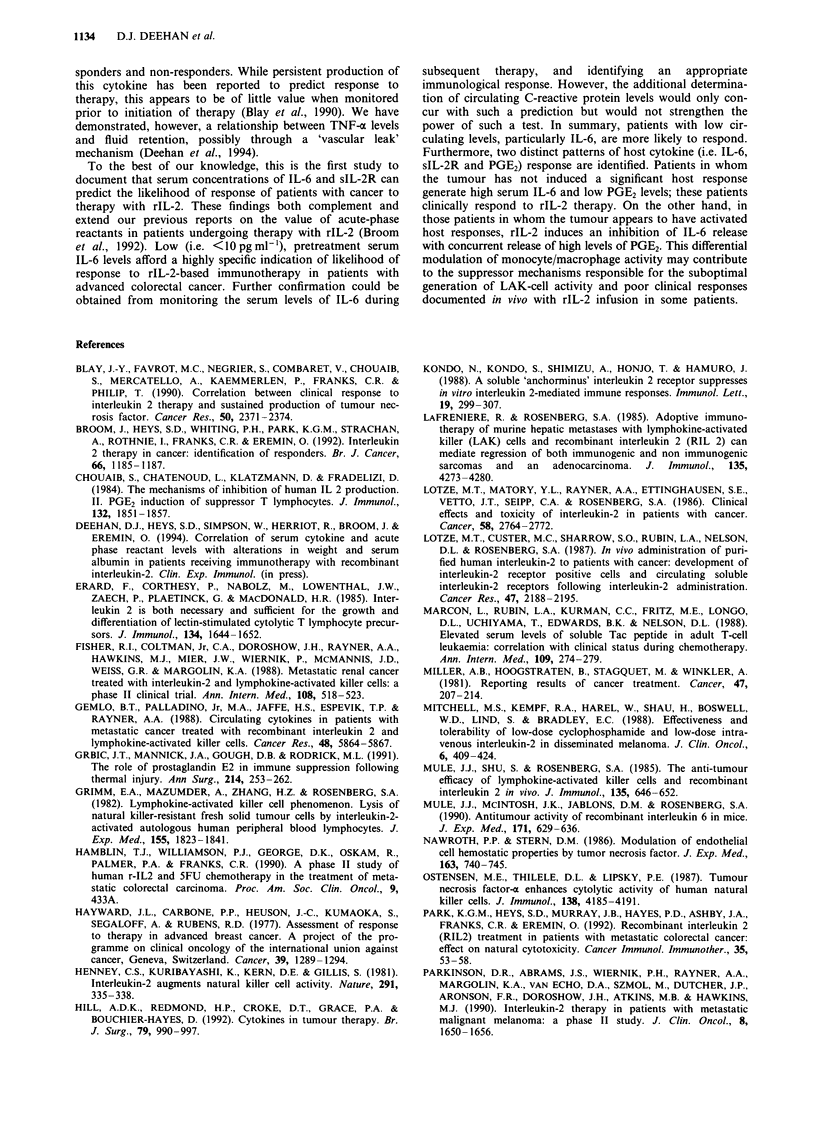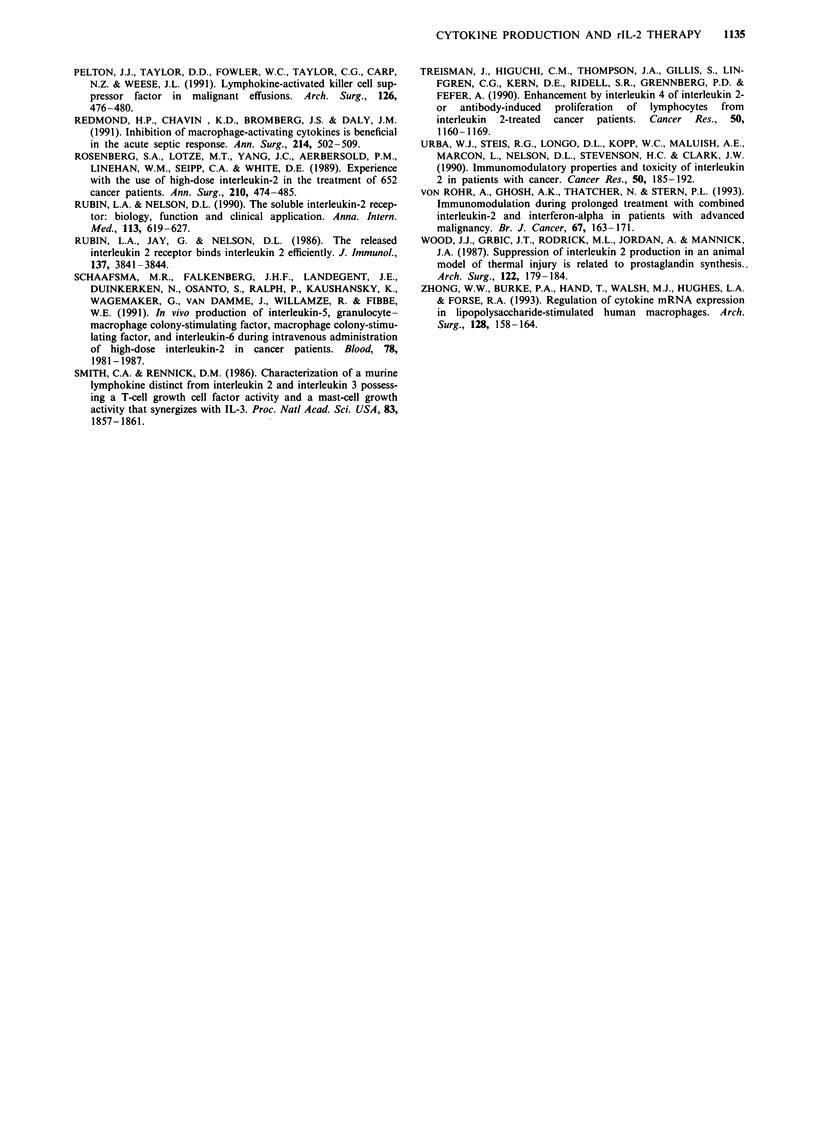# In vivo cytokine production and recombinant interleukin 2 immunotherapy: an insight into the possible mechanisms underlying clinical responses.

**DOI:** 10.1038/bjc.1994.222

**Published:** 1994-06

**Authors:** D. J. Deehan, S. D. Heys, W. G. Simpson, J. Broom, C. Franks, O. Eremin

**Affiliations:** Department of Surgery, University of Aberdeen, Scotland, UK.

## Abstract

Recombinant interleukin 2 (rIL-2), when given to patients with advanced malignant disease, induces a limited beneficial effect, with only 20-30% of patients with solid tumours responding. This present study has identified those patients with advanced colorectal cancer most likely to respond to rIL-2 therapy, by analysis of serum cytokine levels, prior to and during rIL-2 treatment, documented in responders and non-responders. Responders were found to have significantly lower pretreatment serum IL-6 and soluble IL-2 receptor levels (sIL-2R) than non-responders (P < 0.01 and P < 0.05 respectively). During rIL-2 infusion, responders developed high circulating levels of IL-6 and had low constant levels of prostaglandin E2 (PGE2). Non-responders failed to produce IL-6 and demonstrated elevated serum concentrations of PGE2, during infusions of rIL-2. Thus, an enhanced ongoing IL-6 and sIL-2R response, prior to therapy, was detrimental to subsequent treatment with rIL-2. Differential production and/or release of cytokines and prostaglandins, during therapy, further determined the likelihood of response to rIL-2.


Br. J. Cancer (1994), 69, 1130–1135

© Macmillan Press Ltd., 1994

### *In vivo* cytokine production and recombinant interleukin 2 immunotherapy: an insight into the possible mechanisms underlying clinical responses

D.J. Deehan[1], S.D. Heys[1], W.G. Simpson[2], J. Broom[2], C. Franks[3] & O. Eremin[1]

*Departments of [1]Surgery and [2]Biochemistry, University of Aberdeen, Aberdeen, Scotland, UK; [3]Eurocetus, Amsterdam, The Netherlands.*

**Summary** Recombinant interleukin 2 (rIL-2), when given to patients with advanced malignant disease, induces a limited beneficial effect, with only 20–30% of patients with solid tumours responding. This present study has identified those patients with advanced colorectal cancer most likely to respond to rIL-2 therapy, by analysis of serum cytokine levels, prior to and during rIL-2 treatment, documented in responders and non-responders. Responders were found to have significantly lower pretreatment serum IL-6 and soluble IL-2 receptor levels (sIL-2R) than non-responders ($P<0.01$ and $P<0.05$ respectively). During rIL-2 infusion, responders developed high circulating levels of IL-6 and had low constant levels of prostaglandin $E_2$ ($PGE_2$). Non-responders failed to produce IL-6 and demonstrated elevated serum concentrations of $PGE_2$, during infusions of rIL-2. Thus, an enhanced ongoing IL-6 and sIL-2R response, prior to therapy, was detrimental to subsequent treatment with rIL-2. Differential production and/or release of cytokines and prostaglandins, during therapy, further determined the likelihood of response to rIL-2.

Interleukin 2 (IL-2) is a 15.5 kDa T cell-derived cytokine which is integral to the proper activity of the immune system (Erard *et al.*, 1985). It stimulates the activation, proliferation and differentiation of T and B cells, the generation and enhanced cytolytic activity of natural killer (NK) and lymphokine-activated killer (LAK) cells and the generation and release of various cytokines (Henney *et al.*, 1981; Grimm *et al.*, 1982). IL-2 induces, both *in vitro* and *in vivo*, T-cell production of IL-2, IL-4 and gamma-interferon (γ-IFN), and monocyte release of IL-1, tumour necrosis factor (TNF-α) and IL-6, as well as the release of the inhibitory eicosanoid prostaglandin $E_2$ ($PGE_2$) (Schaafsma *et al.*, 1991). Furthermore, IL-2 enhances the expression of the cell-surface receptor for IL-2 (IL-2R) on T and B cells and monocytes (Lotze *et al.*, 1987). Binding of IL-2 to the α-subunit of the receptor (R) results in the release of a 45 kDa cleaved soluble(s) fragment (sIL-2R) (Rubin *et al.*, 1986). sIL-2R is not known to have agonist activity but is capable of binding with low affinity to circulating IL-2 and may, *in vivo*, reduce the bioavailability of exogenously administered IL-2 (Kondon *et al.*, 1988; Rubin *et al.*, 1990).

Early animal experiments demonstrated that exogenously administered IL-2 could induce the regression of a number of experimental tumours (LaFreniere *et al.*, 1985). Recombinant IL-2 (rIL-2) has been given, alone or in combination with other chemotherapeutic agents, to patients with advanced cancers in an attempt to enhance host anti-tumour mechanisms and thus improve survival (Rosenberg *et al.*, 1989). The highest clinical response rates documented to date have been achieved in patients with renal cancer and malignant melanoma (Fisher *et al.*, 1988; Parkinson *et al.*, 1990). However, therapy for patients with other solid tumours, e.g. colorectal cancer, has been disappointing, with maximal clinical response rates of 20–30% being described (Hamblin *et al.*, 1990). The mechanism(s) underlying this tumour selectivity has not been fully characterised but may be dependent, in part, upon the ability of the host to generate an appropriate cytokine response.

The monocyte-derived cytokines IL-1, IL-6 and TNF-α and $PGE_2$ exert regulatory influences upon the immune system. Antigen interaction with monocytes results in the early release of IL-1, TNF-α and IL-6. These cytokines co-stimulate each other's release from the monocyte/macrophage population and also cooperate in a range of immune-enhancing actions, e.g. induction of acute-phase protein release (Zhong *et al.*, 1993). Subsequent monocyte production of $PGE_2$ down-regulates the release of these cytokines and directly inhibits the action of T-helper cells, responsible for the production of IL-2 and IL-4. Sustained imbalances in the release of IL-6 and $PGE_2$ have been found in states of anergy and sepsis (Redmond *et al.*, 1991). The mechanisms responsible for the different patterns of cytokine production by monocytes are unknown. However, appropriate IL-6 release is necessary for the anti-cancer efficacy of rIL-2.

Several cytokines are known to influence the function of the anti-cancer NK and LAK cells (von Rohr *et al.*, 1993). IL-2 and IL-6 are essential co-factors in the generation and optimal cytolytic activity of these cells. $PGE_2$, *in vitro*, can inhibit LAK-cell function, and sIL-2R may limit the availability of IL-2. NK and LAK cells are capable of lysing allogeneic and, in the case of LAK cells, autologous tumour cells, through both MHC- and non-MHC restricted pathways (Mule *et al.*, 1990). In patients with advanced colorectal cancer, we have demonstrated that continuous administration of rIL-2 results in maximum *in vivo* stimulation of NK activity but suboptimal LAK cell cytotoxicity (Park *et al.*, 1992). The latter cells, however, could undergo maximum stimulation with further *in vitro* incubation with rIL-2. IL-2 may therefore concurrently generate increased suppressor mechanisms and/or activity through the release of certain endogenous humoral mediators, e.g. $PGE_2$ (Pelton *et al.*, 1991).

The aim of our study was therefore to define more precisely the pattern of cytokine (IL-4, IL-6, TNF-α, sIL-2R) and prostaglandin ($PGE_2$) release in patients with solid cancers, prior to and during therapy. The patterns documented offer possible explanations for the differential effect on anti-cancer host defences and selective anti-tumour efficacy of rIL-2 therapy.

### Patients and methods

### *Patients*

Twenty patients with metastatic or locally advanced colorectal carcinoma (Dukes C or D) were studied. All patients had an ambulatory performance status (Eastern Cooperative

Correspondence: D.J. Deehan, Research Fellow, Department of Surgery, Medical School Buildings, Polwarth Building, Aberdeen AB9 2ZD, UK.

Received 26 November 1993; and in revised form 14 January 1994.

Oncology Group 0–1, Karnofsky >80%) with a life expectancy greater than 3 months. Liver and renal function tests were within normal limits and patelet and white cell counts were above $120 \times 10^9 l^{-1}$ and $3 \times 10^9 l^{-1}$ respectively. No patient had received systemic chemotherapy, radiotherapy or immunotherapy for the 4 weeks prior to the rIL-2 infusion. All patients gave written informed consent to participate in the study, which was part of a prospective, randomised, European study, and had been approved by the Joint Ethical Committee of Grampian Health Board and Aberdeen University. Patients were monitored in a surgical high-dependency unit and hourly recordings of pulse, blood pressure, temperature and urine output were documented.

*Dosage of rIL-2*

A constant intravenous infusion of rIL-2 (Proleukin, Eurocetus Corporation, Amsterdam, The Netherlands) was administered for 5 days (120 h). Dosage was calculated according to the schedule, $18 \times 10^6$ IU $m^{-2}$ 24 $h^{-1}$ for the total of 120 h. No significant alteration in rate of infusion was required because of severe toxicity. Forty-eight hours following cessation of the rIL-2 infusion, patients received a bolus intravenous injection of 5-fluorouracil (600 mg $m^{-2}$) and folinic acid (25 mg $m^{-2}$), the chemotherapy being repeated weekly for 3 weeks. At the end of this 4 week period, constituting a cycle, patients were reassessed, and if there was evidence of stasis or a clinical tumour response to the disease, patients received further cycles up to a maximum of 6. However, if there was clear evidence of progression of disease, no further immunotherapy was given.

*Timing of sample collection and purification*

Serum was collected from all patients at the start (prior to infusion) and end of the rIL-2 administration (120 h) at all cycles. Peripheral blood was collected without the use of a tourniquet. In a further ten patients, serum was collected 0, 12, 24, 48, 72, 96 and 120 h from the start of the infusion during the first cycle. Blood was allowed to clot and then spun at 1,000 *g* for 10 min. Serum was removed and stored at −80°C until required for analysis. For the analysis of plasma levels of $PGE_2$, blood was collected in EDTA tubes in the presence of the cyclo-oxygenase inhibitor indomethacin [1% (w/v) final concentration] and stored at −80°C, at pH 4.5, titrated by the addition of acetic acid. IL-4, IL-6, sIL-2R and TNF-α were assayed directly from the stored serum. Plasma for $PGE_2$ was purified by passage through Sep-Pak $C_{18}$ columns and eluting the sample with methanol.

*Cytokine analysis*

Interleukin 4, IL-6 and TNF-α were measured by an enzyme-linked immunosorbent assay (ELISA) using the 'sandwich' technique (Quantikine, British Biotechnology, Abington, UK). Minimum detectable serum levels were 31.3 pg $ml^{-1}$, 3.13 pg $ml^{-1}$ and 15.7 pg $ml^{-1}$ respectively. The coefficient of variation (CV) of the assays was found to be less than 3%. Soluble IL-2R (CD45) was measured using an ELISA plate from Dako (Dakopatts, Glostrup, Denmark). The minimum detectable serum level of sIL-2R was 16 U $ml^{-1}$ with a plate CV of 4%. $PGE_2$ was measured using an ELISA plate with a minimum detectable level of 2 pg $ml^{-1}$ (Biotrak, Amersham Life Science, UK). Highest cross-reactivity was for $PGE_1$ at 7%. The CV of the plate was found to be 3%. In each assay, a standard curve was constructed and all samples were assayed in duplicate.

*Response criteria*

Clinically palpable disease (e.g. lymphadenopathy in neck) was evaluated by careful caliper measurements. Impalpable disease (e.g. pulmonary and hepatic metastases) was assessed using a variety of modalities – ultrasonography, computerised tomography and magnetic resonance imaging. Appropriate plain radiography of selected anatomical areas was also carried out. Using standard UICC criteria (Hayward *et al.*, 1977; Miller *et al.*, 1981), a complete response (CR) was defined as the absolute disappearance of all clinically detectable disease for at least 4 weeks. A partial response was defined as a 50% or greater reduction in total tumour mass as measured by the sum of the products of the two longest perpendicular diameters of all measurable lesions. This status had to remain unchanged for 4 weeks, with no simultaneous progression of assessable disease or development of new lesions. Stable disease (SD) was defined as a less than 25% increase or a less than 50% decrease in total tumour size. Progressive disease (PD) was defined as a 25% or greater increase in tumour mass or the appearance of new lesions.

*Statistical analysis*

Statistical analyses were done using non-parametric tests. Grouped data were analysed with the Mann–Whitney *U*-test. Serial recordings of grouped data are expressed as median and interquartile range. Analysis of consecutive dependent data was performed using ANOVA. All *P*-values are two-tailed.

### Results

*Patient characteristics*

Twenty patients were studied, ten females and ten males. The age range was 36–71 years (median 63). No patient underwent a complete response. Seven exhibited a partial response (responders), seven had stasis of disease and six patients had progressive disease (classified as non-responders). There was no sex difference in those patients who responded (three males, four females). Non-responders were classified as those patients who had either stasis or progression of disease.

*Pretreatment IL-6 and sIL-2R levels and response to rIL-2*

Pretreatment serum IL-6 levels in the seven responders were all less than 10 pg $ml^{-1}$ (range 3–8 pg $ml^{-1}$; median 5 pg $ml^{-1}$) (Figure 1). In the 13 patients who did not respond to therapy, the basal IL-6 level varied widely (range 6–151 pg $ml^{-1}$; median 24 pg $ml^{-1}$). Those patients who responded to rIL-2 therapy had low (<10 pg $ml^{-1}$) pretreatment serum IL-6 values compared with non-responders (100% of responders *vs* 8% of non-responders) ($U = 5$, $P < 0.001$). Basal serum levels of sIL-2R (Figure 1), in those patients who responded to treatment, were significantly lower (range 250–675 IU $ml^{-1}$; median 430 IU $ml^{-1}$) than in the non-responders (range 360–5,520 IU $ml^{-1}$; median 1,190 IU $ml^{-1}$) (100% of responders *vs* 46% of non-responders) ($U = 14$, $P < 0.05$).

**Figure 1** Influence of pretreatment serum IL-6 and sIL-2R concentration upon likelihood of response. **a**, Responders ($n = 7$); **b**, non-responders ($n = 13$).

*IL-6 levels during repeated cycles of rIL-2*

IL-6 was assayed at the beginning and end of each 120 h infusion of rIL-2. In six of the seven patients who responded to therapy, there was a consistent rise in serum IL-6 at the end of each cycle (Figure 2). Values then returned to pretreatment levels at the start of the next cycle. This pattern was repeated with all subsequent cycles of rIL-2 infusion in these patients. In one patient who responded clinically and received five cycles of rIL-2 there was no change in serum level of this cytokine during the infusions. In the 13 non-responders, no such pattern was identified. Only one patient exhibited an elevation in IL-6 (during the first infusion). Otherwise, serum IL-6 levels remained unchanged or fell progressively.

*sIl-2R levels during repeated cycles of rIL-2*

All seven responders exhibited elevations in sIL-2R at the end of each infusion. Peak values ranged from 4,240 to 6,160 IU ml$^{-1}$ (median 5,620 IU ml$^{-1}$). Serum levels then fell, but always remained above pretreatment values at the start of the next cycle. One patient continued to have significantly elevated serum levels of the receptor throughout all five cycles. This pattern of potentiation was seen in 10 of the 13 non-responders (data not shown). Three exhibited no variation from baseline levels in serum concentrations at the end of the infusion. Maximum levels attained in this group were similar to those documented in the responders. In both groups of patients, no decrease in peak values attained at 120 h was found.

*IL-6 levels during the first rIL-2 infusion*

Serum IL-6 was determined in ten patients (three responders, seven non-responders) during the first 120 h infusion of rIL-2 (Figure 3). As described above, basal levels in the non-responders were significantly higher than in the responders. These high values then fell progressively throughout the infusion (120 h: 27 ± 5 pg ml$^{-1}$). By contrast, in the responder group, low basal serum concentrations rose to a peak at the end of the infusion (120 h: 97 ± 19 pg ml$^{-1}$).

*sIL-2R levels during the first rIL-2 infusion*

Serum levels of sIL-2R rose progressively in both groups of patients to peak at the end of the infusion (responders, 5,380 ± 450 IU ml$^{-1}$; non-responders, 4,570 ± 520 IU ml$^{-1}$). Other than basal values (see above) no significant differences were identified in the magnitude or timing of the increase in serum levels of the receptor in the two patient groups. A stimulation index of the value at time *t* compared with basal value for the two groups of patients was also carried out (Figure 4). In the responding group of patients, levels were significantly elevated compared with the non-responding patients by 24 h and remained so until 120 h ($P < 0.01$). A peak stimulation index of 15 was achieved in the responding group, whereas that for the non-responding group was only 1.87.

*$PGE_2$ during rIL-2 infusion*

Basal serum concentrations of $PGE_2$ were not significantly different for the two groups (range 15–63 pg ml$^{-1}$). In the responder group, no significant alterations in serum levels occurred during the infusions (Figure 5). The serum $PGE_2$ concentration rose progressively throughout the infusions to peak at 72 h (235 ± 25 pg ml$^{-1}$) and remained elevated until 120 h. Significant differences in serum level between the two groups were attained by 48 h from commencement of the infusions ($P < 0.05$). These differences persisted for the remainder of the rIL-2 infusions (48–120 h).

**Figure 2** Serum concentrations of IL-6 in responders during repeated infusions of rIL-2. Results expressed as median ± interquartile range. D0, day 0; D5, day 5 of each 120 h rIL-2 infusion of each cycle (C).

**Figure 3** Serum IL-6 concentrations for responders during first infusion of rIL-2. Median ± interquartile range. *$P < 0.05$, Mann–Whitney *U*-test (times 0, 24, 72, 96 and 120 h). **a**, Responders; **b**, non-responders.

**Figure 4** Stimulation index for serum sIL-2R concentrations in responders and non-responders during first infusion of rIL-2. Median ± interquartile range. **$P < 0.01$, Mann–Whitney *U*-test (time 0, 24 h). **a**, Responders; **b**, non-responders.

**Figure 5** Serum concentrations of $PGE_2$ for responders and non-responders during first infusion of rIL-2. Median ± interquartile range. *$P < 0.05$, Mann–Whitney *U*-test (times 48, 72, 96 and 120 h). **a**, Responders; **b**, non-responders.

*IL-4 and TNF-α during rIL-2 infusion*

IL-4 was not detected in any serum sample obtained from either group of patients (responders/non-responders). Serum levels of TNF-α were assayed in ten patients (three responders, seven non-responders). Pretreatment serum TNF-α levels were all below detectable limits. In all but one patient serum concentrations rose by 48 h of the infusion to detectable levels. Maximum values were achieved in these nine patients by 92 h and the range was 80–220 pg $ml^{-1}$. However, no differences in either peak value attained or the time taken to achieve peak values were found between the two groups of patients. The one patient who exhibited no serum TNF-α response was a non-responder.

### Discussion

Recombinant IL-2 has been administered to patients with advanced cancer in an effort to enhance anti-cancer host defence mechanisms and thus induce remissions (Mule *et al.*, 1985). Both enhanced *in vivo* NK- and LAK-cell cytolytic activities and appropriate monocyte-derived cytokine release have been postulated as important anti-cancer mechanisms (Gemlo *et al.*, 1988; von Rohr *et al.*, 1993). While it is recognised that NK- and LAK-cell activities correlate with tumour responses in animal studies, this is less well established in man (Mitchell *et al.*, 1988). LAK-cell activity, *in vivo*, has been found to be suboptimal in patients with advanced colorectal cancer receiving continuous infusions of rIL-2 (Park *et al.*, 1992). Maximal activity of the LAK cells, however, could be achieved by further *in vitro* incubation with rIL-2. Pre-existing, or therapy-induced, circulating suppressor factors appeared to be modifying host responses to exogenous rIL-2. Imbalance in the production of the immune-enhancing cytokines IL-1, IL-6 and TNF-α and the inhibitory eicosanoid $PGE_2$ may also modify the host anti-cancer response to rIL-2.

While much is known of the cytokine regulatory influences upon NK- and LAK-cell function, the relevance of the therapy-induced generation of cytokines, *in vivo*, is poorly understood. IL-6, TNF-α, sIL-2R and γ-IFN have all been detected sporadically in the sera of patients receiving immunotherapy (Gemlo *et al.*, 1988; Urba *et al.*, 1990). However, the significance of these *in vivo* findings in relation to anti-cancer effects is unclear at present. IL-6 promotes NK- and LAK-cell generation and activity. It may also directly mediate some of the anti-cancer effects of IL-2. TNF-α enhances NK- and LAK-cell activity, as well as inducing the disruption of tumour vascular architecture through release of endonucleases and proteases (Nawroth & Stern, 1986; Ostensen *et al.*, 1987). Sustained elevations in serum TNF-α have been correlated with response to rIL-2 therapy (Blay *et al.*, 1990). The soluble IL-2 receptor is shed from the membrane-bound p55 α-chain after ligand binding. Circulating levels are therefore believed to be representative of overall IL-2 activity, although cleavage of the solitary α-chain does not result in signal transduction (Marcon *et al.*, 1988). IL-4 is a T cell-derived cytokine with pleiotropic effects upon the immune system (Hill *et al.*, 1992). It stimulates the growth of activated T, B and NK cells. It also enhances cytolytic T-cell activity, yet paradoxically reduces LAK-cell killing (Smith *et al.*, 1986; Treisman *et al.*, 1990). Activated monocytes have been shown to down-regulate the activity of these cytokines, through the release of $PGE_2$ (Chouaib *et al.*, 1984; Wood *et al.*, 1987). $PGE_2$ inhibits monocyte release of IL-1 and TNF-α, and the generation and activity of NK and LAK cells (Grbic *et al.*, 1991).

In this study, patients with advanced colorectal cancer undergoing therapy with rIL-2 and chemotherapy were evaluated. We have documented that patients with demonstrable anti-tumour responses (responders) were more likely to have low basal levels of both IL-6 and sIL-2R. Our results suggest that in these patients there is an absence of an ongoing immune, possibly anti-cancer, response and low basal activity of cell-mediated immunity. In the non-responders, on the other hand, there was a wide range of basal levels of these cytokines. Very few (8%) had normal serum IL-6 levels, while almost half (46%) had high levels of sIL-2R, suggesting an ongoing immune response, albeit ineffectual against advanced cancers. Although the subsequent augmented IL-6 response to rIL-2 therapy was highly predictive (90%) of a beneficial anti-tumour response in the first course of treatment, the sIL-2R levels bore no relationship to the clinical responses documented. High basal levels of IL-6 suggest the presence of a substantial population of activated monocytes/macrophages responding to stimulatory signals. Likewise, high basal levels of serum sIL-2R are the result of increased serum concentrations of the cleaved α-chain of the IL-2 receptor. Very high circulating levels of IL-6 and sIL-2R are also found in patients with sepsis and active autoimmune disease (e.g. rheumatoid arthritis), confirming the postulate that IL-6 and sIL-2R are sensitive barometers of perturbations of cell-mediated immunity.

A clear difference in the pattern of IL-6 production was seen between these two groups of patients. In the responders, IL-6 levels were always much higher at the end of the 5 day infusion, with return to the pretreatment range at the start of the subsequent infusion. This biorhythmic response was seen in all the cycles of rIL-2 therapy. This implies a consistent production of immune-enhancing cytokines in response to exogenous rIL-2 and stimulation of the cells of the lymphoreticular system. In contrast, no such pattern was seen in the non-responders. Lack of generation of high levels of IL-6 in response to rIL-2 is potentially detrimental and may limit maximal NK/LAK-cell generation. This would be in keeping with our previous data demonstrating suboptimal LAK-cell activity in patients receiving rIL-2 therapy (Park *et al.*, 1992). No difference was seen in the serum levels of sIL-2R during the infusions in the two groups of patients. Circulating sIL-2R levels increased in all responders and in the majority of non-responders at the end of each 5 day infusion. Increased binding of rIL-2 to the α-chain of the IL-2 receptor may be occurring here. However, it is uncertain whether this is part of the low-affinity isolated p55 receptor which does not transduce signals or a fragment of the high-affinity α/β-heterodimer. These very high levels of sIL-2R in the serum may also reflect decreased urinary excretion secondary to rIL-2-induced renal tubular dysfunction (Lotze *et al.*, 1986). Our data identify no differential pattern in absolute levels, during therapy, of serum concentrations of sIL-2R between responders and non-responders. This contradicts the view that sIL-2R may bind to exogenously administered rIL-2 and thus reduce its efficacy. If that were the case, clinical response would be associated with lower circulating levels of this receptor. However, such binding of IL-2 to the Tac fragment, to our knowledge, has only been demonstrated *in vitro* and as such may not be representative of *in vivo* physiology (Rubin *et al.*, 1986). Such binding of the sIL-2R fragment to rIL-2 that does occur in the circulation appears not to influence the efficacy of rIL-2.

Analysis of the serum cytokine concentrations during the first infusion revealed two distinct patterns of cytokine production. In those patients who responded to therapy, there was progressive elevation in the serum IL-6 with no change in serum $PGE_2$ concentration. In non-responders, on the other hand, serum IL-6 concentrations fell with correspondingly increasing levels of $PGE_2$ during the rIL-2 infusion. A finely balanced interaction between the stimulatory IL-6 and the inhibitory $PGE_2$ thus may determine the effect on anti-cancer defences and the response to therapy. No intra-infusion difference in levels of sIL-2R was seen. Lack of detectable IL-4 at any time point is open to various interpretations; *in situ* generation and rapid utilisation may partly explain the undetectable levels. We have also analysed the serum levels of TNF-α in our patients during the rIL-2 infusion. Serum levels rose in nine of the ten patients studied. Peak levels were attained at 72 h from the commencement of infusion and were of the order of 100–150 pg $ml^{-1}$. However, no differential TNF-α release was seen between responders and non-responders. While persistent production of this cytokine has been reported to predict response to therapy, this appears to be of little value when monitored prior to initiation of therapy (Blay *et al*., 1990). We have demonstrated, however, a relationship between TNF-α levels and fluid retention, possibly through a 'vascular leak' mechanism (Deehan *et al*., 1994).

To the best of our knowledge, this is the first study to document that serum concentrations of IL-6 and sIL-2R can predict the likelihood of response of patients with cancer to therapy with rIL-2. These findings both complement and extend our previous reports on the value of acute-phase reactants in patients undergoing therapy with rIL-2 (Broom *et al*., 1992). Low (i.e. $<10\ pg\ ml^{-1}$), pretreatment serum IL-6 levels afford a highly specific indication of likelihood of response to rIL-2-based immunotherapy in patients with advanced colorectal cancer. Further confirmation could be obtained from monitoring the serum levels of IL-6 during subsequent therapy, and identifying an appropriate immunological response. However, the additional determination of circulating C-reactive protein levels would only concur with such a prediction but would not strengthen the power of such a test. In summary, patients with low circulating levels, particularly IL-6, are more likely to respond. Furthermore, two distinct patterns of host cytokine (i.e. IL-6, sIL-2R and $PGE_2$) response are identified. Patients in whom the tumour has not induced a significant host response generate high serum IL-6 and low $PGE_2$ levels; these patients clinically respond to rIL-2 therapy. On the other hand, in those patients in whom the tumour appears to have activated host responses, rIL-2 induces an inhibition of IL-6 release with concurrent release of high levels of $PGE_2$. This differential modulation of monocyte/macrophage activity may contribute to the suppressor mechanisms responsible for the suboptimal generation of LAK-cell activity and poor clinical responses documented *in vivo* with rIL-2 infusion in some patients.

### References

BLAY, J.-Y., FAVROT, M.C., NEGRIER, S., COMBARET, V., CHOUAIB, S., MERCATELLO, A., KAEMMERLEN, P., FRANKS, C.R. & PHILIP, T. (1990). Correlation between clinical response to interleukin 2 therapy and sustained production of tumour necrosis factor. *Cancer Res*., **50**, 2371–2374.

BROOM, J., HEYS, S.D., WHITING, P.H., PARK, K.G.M., STRACHAN, A., ROTHNIE, I., FRANKS, C.R. & EREMIN, O. (1992). Interleukin 2 therapy in cancer: identification of responders. *Br. J. Cancer*, **66**, 1185–1187.

CHOUAIB, S., CHATENOUD, L., KLATZMANN, D. & FRADELIZI, D. (1984). The mechanisms of inhibition of human IL 2 production. II. $PGE_2$ induction of suppressor T lymphocytes. *J. Immunol*., **132**, 1851–1857.

DEEHAN, D.J., HEYS, S.D., SIMPSON, W., HERRIOT, R., BROOM, J. & EREMIN, O. (1994). Correlation of serum cytokine and acute phase reactant levels with alterations in weight and serum albumin in patients receiving immunotherapy with recombinant interleukin-2. *Clin. Exp. Immunol*. (in press).

ERARD, F., CORTHESY, P., NABOLZ, M., LOWENTHAL, J.W., ZAECH, P., PLAETINCK, G. & MACDONALD, H.R. (1985). Interleukin 2 is both necessary and sufficient for the growth and differentiation of lectin-stimulated cytolytic T lymphocyte precursors. *J. Immunol*., **134**, 1644–1652.

FISHER, R.I., COLTMAN, Jr, C.A., DOROSHOW, J.H., RAYNER, A.A., HAWKINS, M.J., MIER, J.W., WIERNIK, P., MCMANNIS, J.D., WEISS, G.R. & MARGOLIN, K.A. (1988). Metastatic renal cancer treated with interleukin-2 and lymphokine-activated killer cells: a phase II clinical trial. *Ann. Intern. Med*., **108**, 518–523.

GEMLO, B.T., PALLADINO, Jr, M.A., JAFFE, H.S., ESPEVIK, T.P. & RAYNER, A.A. (1988). Circulating cytokines in patients with metastatic cancer treated with recombinant interleukin 2 and lymphokine-activated killer cells. *Cancer Res*., **48**, 5864–5867.

GRBIC, J.T., MANNICK, J.A., GOUGH, D.B. & RODRICK, M.L. (1991). The role of prostaglandin E2 in immune suppression following thermal injury. *Ann Surg*., **214**, 253–262.

GRIMM, E.A., MAZUMDER, A., ZHANG, H.Z. & ROSENBERG, S.A. (1982). Lymphokine-activated killer cell phenomenon. Lysis of natural killer-resistant fresh solid tumour cells by interleukin-2-activated autologous human peripheral blood lymphocytes. *J. Exp. Med*., **155**, 1823–1841.

HAMBLIN, T.J., WILLIAMSON, P.J., GEORGE, D.K., OSKAM, R., PALMER, P.A. & FRANKS, C.R. (1990). A phase II study of human r-IL2 and 5FU chemotherapy in the treatment of metastatic colorectal carcinoma. *Proc. Am. Soc. Clin. Oncol*., **9**, 433A.

HAYWARD, J.L., CARBONE, P.P., HEUSON, J.-C., KUMAOKA, S., SEGALOFF, A. & RUBENS, R.D. (1977). Assessment of response to therapy in advanced breast cancer. A project of the programme on clinical oncology of the international union against cancer, Geneva, Switzerland. *Cancer*, **39**, 1289–1294.

HENNEY, C.S., KURIBAYASHI, K., KERN, D.E. & GILLIS, S. (1981). Interleukin-2 augments natural killer cell activity. *Nature*, **291**, 335–338.

HILL, A.D.K., REDMOND, H.P., CROKE, D.T., GRACE, P.A. & BOUCHIER-HAYES, D. (1992). Cytokines in tumour therapy. *Br. J. Surg*., **79**, 990–997.

KONDO, N., KONDO, S., SHIMIZU, A., HONJO, T. & HAMURO, J. (1988). A soluble 'anchorminus' interleukin 2 receptor suppresses *in vitro* interleukin 2-mediated immune responses. *Immunol. Lett*., **19**, 299–307.

LAFRENIERE, R. & ROSENBERG, S.A. (1985). Adoptive immunotherapy of murine hepatic metastases with lymphokine-activated killer (LAK) cells and recombinant interleukin 2 (RIL 2) can mediate regression of both immunogenic and non immunogenic sarcomas and an adenocarcinoma. *J. Immunol*., **135**, 4273–4280.

LOTZE, M.T., MATORY, Y.L., RAYNER, A.A., ETTINGHAUSEN, S.E., VETTO, J.T., SEIPP, C.A. & ROSENBERG, S.A. (1986). Clinical effects and toxicity of interleukin-2 in patients with cancer. *Cancer*, **58**, 2764–2772.

LOTZE, M.T., CUSTER, M.C., SHARROW, S.O., RUBIN, L.A., NELSON, D.L. & ROSENBERG, S.A. (1987). *In vivo* administration of purified human interleukin-2 to patients with cancer: development of interleukin-2 receptor positive cells and circulating soluble interleukin-2 receptors following interleukin-2 administration. *Cancer Res*., **47**, 2188–2195.

MARCON, L., RUBIN, L.A., KURMAN, C.C., FRITZ, M.E., LONGO, D.L., UCHIYAMA, T., EDWARDS, B.K. & NELSON, D.L. (1988). Elevated serum levels of soluble Tac peptide in adult T-cell leukaemia: correlation with clinical status during chemotherapy. *Ann. Intern. Med*., **109**, 274–279.

MILLER, A.B., HOOGSTRATEN, B., STAGQUET, M. & WINKLER, A. (1981). Reporting results of cancer treatment. *Cancer*, **47**, 207–214.

MITCHELL, M.S., KEMPF, R.A., HAREL, W., SHAU, H., BOSWELL, W.D., LIND, S. & BRADLEY, E.C. (1988). Effectiveness and tolerability of low-dose cyclophosphamide and low-dose intravenous interleukin-2 in disseminated melanoma. *J. Clin. Oncol*., **6**, 409–424.

MULE, J.J., SHU, S. & ROSENBERG, S.A. (1985). The anti-tumour efficacy of lymphokine-activated killer cells and recombinant interleukin 2 *in vivo*. *J. Immunol*., **135**, 646–652.

MULE, J.J., MCINTOSH, J.K., JABLONS, D.M. & ROSENBERG, S.A. (1990). Antitumour activity of recombinant interleukin 6 in mice. *J. Exp. Med*., **171**, 629–636.

NAWROTH, P.P. & STERN, D.M. (1986). Modulation of endothelial cell hemostatic properties by tumor necrosis factor. *J. Exp. Med*., **163**, 740–745.

OSTENSEN, M.E., THILELE, D.L. & LIPSKY, P.E. (1987). Tumour necrosis factor-α enhances cytolytic activity of human natural killer cells. *J. Immunol*., **138**, 4185–4191.

PARK, K.G.M., HEYS, S.D., MURRAY, J.B., HAYES, P.D., ASHBY, J.A., FRANKS, C.R. & EREMIN, O. (1992). Recombinant interleukin 2 (RIL2) treatment in patients with metastatic colorectal cancer: effect on natural cytotoxicity. *Cancer Immunol. Immunother*., **35**, 53–58.

PARKINSON, D.R., ABRAMS, J.S., WIERNIK, P.H., RAYNER, A.A., MARGOLIN, K.A., VAN ECHO, D.A., SZMOL, M., DUTCHER, J.P., ARONSON, F.R., DOROSHOW, J.H., ATKINS, M.B. & HAWKINS, M.J. (1990). Interleukin-2 therapy in patients with metastatic malignant melanoma: a phase II study. *J. Clin. Oncol*., **8**, 1650–1656.

PELTON, J.J., TAYLOR, D.D., FOWLER, W.C., TAYLOR, C.G., CARP, N.Z. & WEESE, J.L. (1991). Lymphokine-activated killer cell suppressor factor in malignant effusions. *Arch. Surg.*, **126**, 476–480.

REDMOND, H.P., CHAVIN , K.D., BROMBERG, J.S. & DALY, J.M. (1991). Inhibition of macrophage-activating cytokines is beneficial in the acute septic response. *Ann. Surg.*, **214**, 502–509.

ROSENBERG, S.A., LOTZE, M.T., YANG, J.C., AERBERSOLD, P.M., LINEHAN, W.M., SEIPP, C.A. & WHITE, D.E. (1989). Experience with the use of high-dose interleukin-2 in the treatment of 652 cancer patients. *Ann. Surg.*, **210**, 474–485.

RUBIN, L.A. & NELSON, D.L. (1990). The soluble interleukin-2 receptor: biology, function and clinical application. *Anna. Intern. Med.*, **113**, 619–627.

RUBIN, L.A., JAY, G. & NELSON, D.L. (1986). The released interleukin 2 receptor binds interleukin 2 efficiently. *J. Immunol.*, **137**, 3841–3844.

SCHAAFSMA, M.R., FALKENBERG, J.H.F., LANDEGENT, J.E., DUINKERKEN, N., OSANTO, S., RALPH, P., KAUSHANSKY, K., WAGEMAKER, G., VAN DAMME, J., WILLAMZE, R. & FIBBE, W.E. (1991). *In vivo* production of interleukin-5, granulocyte–macrophage colony-stimulating factor, macrophage colony-stimulating factor, and interleukin-6 during intravenous administration of high-dose interleukin-2 in cancer patients. *Blood*, **78**, 1981–1987.

SMITH, C.A. & RENNICK, D.M. (1986). Characterization of a murine lymphokine distinct from interleukin 2 and interleukin 3 possessing a T-cell growth cell factor activity and a mast-cell growth activity that synergizes with IL-3. *Proc. Natl Acad. Sci. USA*, **83**, 1857–1861.

TREISMAN, J., HIGUCHI, C.M., THOMPSON, J.A., GILLIS, S., LINFGREN, C.G., KERN, D.E., RIDELL, S.R., GRENNBERG, P.D. & FEFER, A. (1990). Enhancement by interleukin 4 of interleukin 2- or antibody-induced proliferation of lymphocytes from interleukin 2-treated cancer patients. *Cancer Res.*, **50**, 1160–1169.

URBA, W.J., STEIS, R.G., LONGO, D.L., KOPP, W.C., MALUISH, A.E., MARCON, L., NELSON, D.L., STEVENSON, H.C. & CLARK, J.W. (1990). Immunomodulatory properties and toxicity of interleukin 2 in patients with cancer. *Cancer Res.*, **50**, 185–192.

VON ROHR, A., GHOSH, A.K., THATCHER, N. & STERN, P.L. (1993). Immunomodulation during prolonged treatment with combined interleukin-2 and interferon-alpha in patients with advanced malignancy. *Br. J. Cancer*, **67**, 163–171.

WOOD, J.J., GRBIC, J.T., RODRICK, M.L., JORDAN, A. & MANNICK, J.A. (1987). Suppression of interleukin 2 production in an animal model of thermal injury is related to prostaglandin synthesis.. *Arch. Surg.*, **122**, 179–184.

ZHONG, W.W., BURKE, P.A., HAND, T., WALSH, M.J., HUGHES, L.A. & FORSE, R.A. (1993). Regulation of cytokine mRNA expression in lipopolysaccharide-stimulated human macrophages. *Arch. Surg.*, **128**, 158–164.